# Examining Discordance in Perceptions of COVID-19 and Influenza Vaccine Safety During Pregnancy Among a Cohort of US Adults

**DOI:** 10.3390/vaccines14030274

**Published:** 2026-03-20

**Authors:** Rachael Piltch-Loeb, Bai Xi Jasmine Chan, Josefina Nuñez Sahr, Chloe Teasdale, Sasha Fleary, Kate Penrose, Jenna Sanborn, Subha Balasubramanian, McKaylee Robertson, Angela Parcesepe

**Affiliations:** 1 Institute for Implementation Science in Population Health, Graduate School of Public Health and Health Policy, City University of New York (CUNY), New York, NY 10027, USA; 2 Department of Environmental, Occupational, Geospatial Health Sciences, City University of New York (CUNY), New York, NY 10027, USA; 3 Department of Epidemiology and Biostatistics, Graduate School of Public Health and Health Policy, City University of New York (CUNY), New York, NY 10027, USA; 4 Department of Community Health and Social Sciences, Graduate School of Public Health and Health Policy, City University of New York (CUNY), New York, NY 10027, USA; 5 Department of Maternal and Child Health, Gillings School of Global Public Health, University of North Carolina at Chapel Hill, Chapel Hill, NC 27599, USA; 6 Carolina Population Center, University of North Carolina at Chapel Hill, Chapel Hill, NC 27599, USA

**Keywords:** COVID-19 vaccine, influenza vaccine, vaccine hesitancy, pregnancy, women’s health

## Abstract

Background/Objectives: Vaccination is an important strategy to protect pregnant people, fetuses, and infants from severe influenza and COVID-19. However, as of April 2025 in the United States, only 14% of pregnant women had received the 2024–2025 COVID-19 vaccine, while influenza vaccine uptake among pregnant women was 38% compared to 57% in 2020. Our study assessed the perceived safety of receiving these vaccines during pregnancy within a community-based national cohort. Methods: Participants reported safety perceptions of vaccination during pregnancy and were categorized as (1) endorsing the safety of both COVID-19 and influenza vaccines, (2) endorsing the safety of only the influenza vaccine, (3) endorsing the safety of only the COVID-19 vaccine and (4) endorsing the safety of neither vaccine during pregnancy. We examined sociodemographics, behaviors, and beliefs correlated with these four categories. Log-binomial models were used to estimate the prevalence ratios of (a) endorsing the safety of only influenza vaccine versus endorsing the safety of both COVID-19 and influenza vaccines during pregnancy, and (b) endorsing safety of neither versus both vaccines during pregnancy. Results: In total, 40% of adults (N = 1725) endorsed the safety of both vaccines during pregnancy, 18% (N = 751) endorsed the safety of only the influenza vaccine, 4% (N = 183) endorsed the safety of only the COVID-19 vaccine, and 38% (N = 1621) did not endorse the safety of either vaccine during pregnancy. Participants who were non-Hispanic Black, reported inconsistent vaccination habits, and expressed low trust in government and healthcare providers were more likely to endorse the safety of only the influenza vaccine or neither vaccine compared with endorsing both vaccines. Conclusions: These findings highlight the need to build trust in the medical system, reduce access barriers, and use equitable, community-driven messaging to strengthen confidence in the safety of receiving the COVID-19 and influenza vaccines during pregnancy.

## 1. Introduction

Seasonal influenza and COVID-19 pose significant health threats during pregnancy [1,2]. Pregnant women are more likely to be hospitalized for influenza compared to non-pregnant women [3]. Influenza infection during pregnancy is linked to an increased risk of stillbirth and may result in preterm birth and low birth weight, especially in severe cases [4,5]. Similarly, people who contract COVID-19 during pregnancy have a greater likelihood of mortality, needing care in the Intensive Care Unit (ICU), and requiring invasive ventilation compared to non-pregnant individuals [6,7].

Vaccination is a critical strategy to protect both mothers and their infants from severe influenza and COVID-19. COVID-19 vaccines during pregnancy have been shown to be highly effective—providing up to 96% protection against infection and 89% against hospitalization, with strong global evidence supporting their safety [8,9]—while influenza vaccines have a longer-established track record of reducing severe respiratory illness [10]. Additionally, both vaccines confer antibodies to infants for up to six months postpartum, offering protection during a period when they are not yet eligible for vaccination [11,12]. Influenza and COVID-19 vaccinations are recommended during pregnancy by the Centers for Disease Control and Prevention (CDC) and the American College of Obstetrics and Gynecology (ACOG) [13,14]. However, as of April 2025, only 14% of pregnant women in the US had received the updated 2024–2025 COVID-19 vaccine [15]. Influenza vaccine uptake among pregnant women was 38%, down from 57% in the same period in 2020 [16]. For both vaccines, vaccination rates reveal disparities among age, race/ethnicity, education, geography, health insurance coverage, and health provider recommendations, among other factors [17]. Furthermore, the proportion of adult women in the US reporting hesitancy towards influenza vaccination during pregnancy increased from 47% in the 2019–2020 period to 60% in the 2022–2023 period [17].

While limited, emerging research suggests that COVID-19 and influenza vaccination uptake during pregnancy may be correlated. A retrospective cohort study (2020–2022) found that receiving the influenza vaccine was positively associated with COVID-19 vaccine acceptance among pregnant women [18]. Perelman et al. [19] found influenza vaccine acceptance among pregnant women to have increased during the COVID-19 pandemic, yet this relationship varied by health insurance status and comorbidities. Similarly, although multiple factors shape vaccine perceptions, national and international evidence consistently demonstrates that peer opinions are an important influence on pregnant individuals’ health decision-making, underscoring that such decisions are rarely made in isolation [20,21,22]. Further research is needed to better understand safety perceptions of both vaccines during pregnancy, as well as how and why safety perceptions of the COVID-19 and influenza vaccines during pregnancy may align or diverge.

Our study aimed to understand safety perceptions of the COVID-19 and influenza vaccines during pregnancy among adults enrolled in a US community-based cohort and among a subset of women of reproductive age. We characterized sociodemographics, behaviors, and beliefs correlated with concordant and discordant perceptions of the safety of COVID-19 and influenza vaccines during pregnancy in order to inform public health efforts to better support vaccination decisions among pregnant women.

## 2. Materials and Methods

### 2.1. Study Population

The Communities, Households, and SARS-CoV-2 Epidemiology (CHASING) COVID Cohort study is a national prospective cohort study launched in March 2020 [23]. Internet-based recruitment strategies were used, such as social media platforms and referrals. Interested participants were asked to provide consent before enrolling. Participants in this community-based sample are from diverse geographic and sociodemographic backgrounds, aged ≥18 years, and residing in the US or US territories. In this fully online cohort study, participants completed quarterly follow-up assessments related to their health and behaviors, symptoms of anxiety and depression, use of mental health services and/or medication, SARS-CoV-2 infection history, and COVID-19 vaccination status from March 2020 to December 2023. Comprehensive details regarding recruitment and follow-up have been presented in previous articles [23,24]. For this analysis, we included all adult participants who (a) completed the baseline assessment and (b) completed the December 2023 assessment, which was the first assessment of beliefs regarding COVID-19 and influenza vaccinations for pregnant women.

#### Ethical Considerations

The study was conducted in accordance with the Declaration of Helsinki, and the protocol was approved by the Institutional Review Board of the City University of New York (CUNY) Graduate School of Public Health and Health Policy (protocol 2020-0256) on 30 March 2020. Participant consent was completed in a web browser on participants’ computers or mobile devices at baseline and at periodic follow-up assessments. Participants could discontinue their participation at any point.

### 2.2. Dependent Variable: Concordance and Discordance of COVID-19 Vaccine Safety and Influenza Vaccine Safety During Pregnancy

Participants’ perceptions on the safety of the COVID-19 and influenza vaccines were collected in the December 2023 assessment. Participants were asked to respond to two statements: “The COVID-19 vaccine is safe to receive during pregnancy” and “The seasonal influenza shot is safe to receive during pregnancy”. Responses were collected on a 5-point Likert scale including the following answers: “strongly agree”, “agree”, “neither agree nor disagree”, “disagree” and “strongly disagree”. Endorsement of the safety of a vaccine during pregnancy was defined by indicating “strongly agree” or “agree” to the corresponding statement. To describe the descriptive outcomes in the overall cohort and among women of reproductive age, responses were combined into four categories: (1) endorsed the safety of both COVID-19 and influenza vaccines during pregnancy, (2) endorsed the safety of only the influenza vaccine during pregnancy, (3) endorsed the safety of only the COVID-19 vaccine during pregnancy and (4) did not endorse the safety of either vaccine during pregnancy. Furthermore, the outcomes for regression models exploring the discordance and concordance of safety perceptions were defined as the (1) endorsement of the safety of only the influenza vaccine during pregnancy and (2) endorsement of the safety of neither vaccine during pregnancy.

### 2.3. Independent Variables

#### 2.3.1. Sociodemographic Characteristics

Between March 2020 and July 2020, baseline data on age, gender, race/ethnicity, education, and annual household income were collected. Data on participants living with any children under 18 years of age in the household was collected up to March 2021. Participants were considered ever pregnant if they reported a pregnancy at any time between March 2020 and December 2023.

#### 2.3.2. Vaccination

Participants were asked to report their COVID-19 and influenza vaccination status and that of household members during follow-up assessments between March 2020 and December 2023. If participants reported receiving at least one COVID-19 booster as of December 2023, they were considered ever boosted against COVID-19. Participants were considered recently vaccinated against influenza if they reported receiving a vaccine between April 2023 and October 2023. The participant’s household was considered ever vaccinated against COVID-19 and/or influenza if at least one household member eligible for vaccination ever reported receiving the respective vaccine during follow-up assessments.

#### 2.3.3. Trusted Sources of COVID-19 Vaccine Information

Participants were asked to identify trusted entities, groups, or individuals for reliable information regarding the COVID-19 vaccine during a follow-up assessment in December 2023. We identified participants who reported trust in “government” and reported trust in “healthcare providers.” Trust in government or intergovernmental entities was defined as endorsing trust in any of the following: the Centers for Disease Control and Prevention (CDC), the World Health Organization (WHO), or the Food and Drug Administration (FDA). Trust in healthcare providers was measured based on participants’ reported trust in their personal physician or other healthcare professional [25].

#### 2.3.4. Healthcare Access

For healthcare access, we examined whether participants had a personal provider and if they reported any barriers to healthcare access. Participants were asked if they had someone they considered their personal doctor or healthcare provider in October 2023. As a measure of barriers to healthcare access, we used factors that affect medical care access, including: not having a personal doctor, concerns about the costs of healthcare, concerns about seeing a doctor due to immigration status, or lack healthcare coverage/insurance [26]. This measure was collected at baseline and dichotomized as any (endorsing 1 or more factors) versus no (endorsing 0 factors) barriers to access [27,28].

#### 2.3.5. Health Literacy

To measure health literacy, participants completed the Single-Item Literacy Screener [29], which asked how often they needed help when reading healthcare information, such as instructions or pamphlets from providers or pharmacies. They were considered health literate if they reported never or rarely (vs. sometimes, often, or always) needing help in the October 2023 survey.

#### 2.3.6. COVID-19 Susceptibility

Susceptibility to severe COVID-19 following SARS-CoV-2 infection was captured at baseline using a composite variable based on several risk factors identified by the CDC [27]. These included any of the following: age of 60 years or older, chronic lung disease (CLD) or chronic obstructive pulmonary disease (COPD), current asthma, diabetes, serious heart conditions (including heart attack, high blood pressure, or angina), kidney disease, immunocompromised status, HIV, or daily smoking. Each factor was coded as present (1) or absent (0), and a cumulative susceptibility score was calculated by summing these binary variables, resulting in a score ranging from 0 to 9. This score was then dichotomized at the median as less (score ≤ 1) versus more (score > 1) susceptible to severe COVID-19.

#### 2.3.7. Perceived Worry About COVID-19 Infection, Illness, or Long-Term Effects

Assessed in December 2023, this measure is dichotomized where participants were considered to be worried about COVID-19 if they indicated they were “somewhat worried” or “very worried” about any of the following three statements: getting sick due to COVID-19, COVID-19 reinfection and/or long-term effects of COVID-19. Participants were considered to be not worried about COVID-19 if they did not endorse being worried about any of the above three statements.

#### 2.3.8. Symptoms of Depression or Anxiety

We examined symptoms of anxiety and depression in December 2023 using two widely used and validated self-administered screening instruments for population-based research [30,31]. Scores of 10 or higher on the Generalized Anxiety Disorder 7-item (GAD-7) questionnaire [30] were categorized as moderate to severe symptoms of anxiety, and scores of 10 or higher on the Patient Health Questionnaire 8-item (PHQ-8) [31] were categorized as moderate to severe symptoms of depression.

### 2.4. Analytic Approach

We present proportions among the overall cohort and the subsample of women of reproductive age (18–49 years old as of April 2020) according to their endorsement of the safety of COVID-19 and influenza vaccines for pregnant women by sociodemographic and health characteristics. Chi-squared tests were used to compare sociodemographic and health characteristics across the four response categories.

Log-binomial models were used to estimate the crude and adjusted prevalence ratios (PRs) of (1) endorsing the safety of only the influenza vaccine during pregnancy compared to endorsing the safety of both vaccines during pregnancy and (2) endorsing the safety of neither vaccine during pregnancy compared to endorsing the safety of both vaccines during pregnancy. Univariable models were run including each of the following characteristics: age, gender, race/ethnicity, education, children under 18 in the household, pregnancy, household annual income, healthcare access, having a personal doctor, having received a COVID-19 booster, having a household member who ever received a COVID-19 vaccination, recent influenza vaccination, having a household member who ever received the influenza vaccination, COVID-19 susceptibility, moderate or severe symptoms of anxiety or depression, trust in government entities or healthcare providers, health literacy, and COVID-19 perceived worry. Age, gender, and education were considered key factors that may be associated with both the outcomes and exposures. Therefore, each exposure variable was examined in separate log-binomial regression models adjusted for age, gender, and education among the overall cohort and the subsample of women of reproductive age. Statistical analyses were conducted using SAS 9.4 (Cary, NC, USA).

## 3. Results

### 3.1. Concordant and Discordant Beliefs

The analytic sample included 4280 adults in the overall cohort and 1532 women of reproductive age (Table 1). In the overall cohort, 40% (n = 1725) endorsed the safety of both vaccines during pregnancy, 38% (n = 1621) did not endorse the safety of either vaccine during pregnancy, 18% (n = 751) endorsed the safety of only the influenza vaccine during pregnancy, and 4% (n = 183) endorsed the safety of only the COVID-19 vaccine during pregnancy. Among women of reproductive age, 43% (n = 656) endorsed the safety of both vaccines, 35% (n = 536) did not endorse the safety of either vaccine, 18% (n = 280) only endorsed the safety of the influenza vaccine and 4% (n = 60) only endorsed the safety of the COVID-19 vaccine during pregnancy.

**Table 1:** Endorsement of the safety of COVID-19 and influenza vaccines among all adults and women of reproductive age in the CHASING COVID Cohort.

	Overall Cohort N = 4280 n (%)	Women of Reproductive Age N = 1532 n (%)
Endorsed the safety of both the COVID-19 and influenza vaccines during pregnancy	1725 (40%)	656 (43%)
Endorsed the safety of neither vaccine during pregnancy	1621 (38%)	536 (35%)
Endorsed the safety of only the influenza vaccine during pregnancy	751 (18%)	280 (18%)
Endorsed the safety of only the COVID-19 vaccine during pregnancy	183 (4%)	60 (4%)

### 3.2. Cohort Characteristics and Perceived Safety of Vaccines During Pregnancy

In the overall cohort, age, race and ethnicity, education, pregnancy status, annual household income, healthcare access barriers, having a personal provider, having received a COVID-19 booster, having a household member who received a COVID-19 vaccination, having received a recent influenza shot, having a household member who received the influenza vaccination, COVID-19 susceptibility, trust in government entities and healthcare providers, and perceived worry about COVID-19 were found to be significantly different across the four outcome groups (Table 2). Higher percentages of participants who endorsed the safety of both vaccines during pregnancy were college graduates (74% vs. 53–62%), those who ever received a COVID-19 vaccine booster (94% vs. 71–85%), those who reported recent influenza vaccination (80% vs. 51–72%), those who were in influenza-vaccinated households (70% vs. 46–64%), and those who reported trust in government entities (87% vs. 65–78%) and in healthcare providers (75% vs. 60–69%) compared to participants who did not endorse the safety of one or both vaccines during pregnancy.

**Table 2:** Participant characteristics and perceived safety of the COVID-19 and influenza vaccines during pregnancy.

	Overall Cohort, n (col%)					Women of Reproductive Age, (col%)				
	Both Vaccines ^1^	Neither Vaccines ^2^	Only Flu Vaccine ^3^	Only COVID-19 Vaccine ^4^	Chi-Sq *p*-Value	Both Vaccines ^1^	Neither Vaccines ^2^	Only Flu Vaccine ^3^	Only COVID-19 Vaccine ^4^	Chi-Sq *p*-Value
	N = 1725 n (col %)	N = 1621 n (col %)	N = 751 n (col %)	N = 183 n (col %)		N = 656 n (col %)	N = 536 n (col %)	N = 280 n (col %)	N = 60 n (col %)	
Age					<0.0001					0.1404
18–29	413 (24%)	300 (19%)	168 (22%)	56 (31%)		254 (39%)	178 (33%)	106 (38%)	28 (47%)	
30–39	548 (32%)	444 (27%)	201 (27%)	43 (24%)		243 (37%)	203 (38%)	109 (39%)	16 (27%)	
40–49	311 (18%)	338 (21%)	121 (16%)	29 (16%)		159 (24%)	155 (29%)	65 (23%)	16 (27%)	
50–64	301 (17%)	352 (22%)	160 (21%)	35 (19%)						
65+	152 (9%)	187 (12%)	101 (13%)	20 (11%)						
Gender					0.7402					
Male	769 (45%)	722 (45%)	318 (42%)	82 (45%)						
Female/non-binary	956 (55%)	899 (55%)	433 (58%)	101 (55%)						
Race/Ethnicity					<0.0001					<0.0001
Hispanic	241 (14%)	315 (19%)	116 (15%)	33 (18%)		110 (17%)	143 (27%)	58 (21%)	12 (20%)	
Non-Hispanic White	1188 (69%)	922 (67%)	468 (62%)	106 (58%)		410 (63%)	230 (43%)	148 (53%)	32 (53%)	
Non-Hispanic Black	115 (7%)	230 (14%)	81 (11%)	26 (14%)		47 (7%)	97 (18%)	30 (11%)	10 (17%)	
Non-Hispanic Asian/PI/other	181 (10%)	154 (10%)	86 (11%)	18 (10%)		89 (14%)	66 (12%)	44 (16%)	6 (10%)	
Education					<0.0001					<0.0001
Less than high school/high school	119 (7%)	246 (15%)	85 (11%)	19 (10%)		51 (8%)	126 (24%)	38 (14%)	7 (12%)	
Some college	322 (19%)	521 (32%)	208 (28%)	50 (27%)		129 (20%)	204 (38%)	83 (30%)	19 (32%)	
College graduate	1284 (74%)	854 (53%)	458 (61%)	114 (62%)		476 (73%)	206 (38%)	159 (57%)	34 (57%)	
Anyone <18 years in household					0.4422					0.003
No	1149 (67%)	1084 (67%)	483 (64%)	128 (70%)		346 (53%)	239 (45%)	122 (44%)	36 (60%)	
Yes	576 (33%)	537 (33%)	268 (36%)	55 (30%)		310 (47%)	297 (55%)	158 (56%)	24 (40%)	
Ever pregnant					<0.0001					0.0014
No	1616 (94%)	1572 (97%)	721 (96%)	175 (96%)		548 (84%)	487 (91%)	250 (89%)	52 (87%)	
Yes	109 (6%)	49 (3%)	30 (4%)	8 (4%)		108 (16%)	49 (9%)	30 (11%)	8 (13%)	
Household annual income					<0.0001					<0.0001
<$50,000/unknown	564 (33%)	779 (48%)	310 (41%)	90 (49%)		228 (35%)	315 (59%)	121 (43%)	35 (58%)	
$50,000–$100,000	549 (32%)	504 (31%)	252 (34%)	50 (27%)		196 (30%)	156 (29%)	90 (32%)	17 (28%)	
>$100,000	612 (35%)	338 (21%)	189 (25%)	43 (24%)		232 (35%)	65 (12%)	69 (25%)	8 (13%)	
Healthcare access					<0.0001					<0.0001
Greater barriers to access	626 (36%)	713 (44%)	268 (36%)	87 (48%)		297 (45%)	306 (57%)	127 (45%)	37 (62%)	
Fewer barriers to access	1099 (64%)	908 (56%)	483 (64%)	96 (52%)		359 (55%)	230 (43%)	153 (55%)	23 (38%)	
Has a personal doctor/provider					<0.0001					<0.0001
No	413 (24%)	523 (32%)	194 (26%)	51 (28%)		177 (27%)	225 (42%)	97 (35%)	19 (32%)	
Yes	1312 (76%)	1098 (68%)	557 (74%)	132 (72%)		479 (73%)	311 (58%)	183 (65%)	41 (68%)	
Ever boosted for COVID-19					<0.0001					<0.0001
No	97 (6%)	463 (29%)	143 (19%)	28 (15%)		58 (9%)	250 (47%)	84 (30%)	12 (20%)	
Yes	1615 (94%)	1116 (71%)	600 (81%)	154 (85%)		598 (91%)	286 (53%)	196 (70%)	48 (80%)	
Has a household member who received a COVID-19 vaccination					<0.0001					<0.0001
No	247 (14%)	451 (28%)	159 (21%)	36 (20%)		75 (11%)	183 (34%)	73 (26%)	11 (18%)	
Yes	1478 (86%)	1170 (72%)	592 (79%)	147 (80%)		581 (89%)	353 (66%)	207 (74%)	49 (82%)	
Recently received the flu vaccine					<0.0001					<0.0001
No	341 (20%)	798 (49%)	211 (28%)	67 (37%)		145 (22%)	351 (66%)	115 (41%)	28 (47%)	
Yes	1384 (80%)	823 (51%)	540 (72%)	116 (63%)		511 (78%)	185 (35%)	165 (59%)	32 (53%)	
Has a household member who ever received a flu vaccination					<0.0001					<0.0001
No	517 (30%)	881 (54%)	268 (36%)	77 (42%)		165 (25%)	303 (57%)	93 (33%)	22 (37%)	
Yes	1208 (70%)	740 (46%)	483 (64%)	206 (58%)		491 (75%)	233 (44%)	187 (67%)	38 (63%)	
Susceptible to COVID-19					0.0004					0.0017
Less susceptible	1409 (82%)	1249 (77%)	563 (75%)	146 (80%)		613 (93%)	467 (87%)	246 (88%)	54 (90%)	
More susceptible	316 (18%)	372 (23%)	188 (25%)	37 (20%)		43 (7%)	69 (13%)	34 (12%)	6 (10%)	
Symptoms of anxiety					0.1043					0.0033
None to mild	1496 (87%)	1372 (85%)	640 (85%)	150 (82%)		550 (84%)	409 (76%)	224 (80%)	43 (72%)	
Moderate/Severe	221 (13%)	246 (15%)	111 (15%)	33 (18%)		104 (16%)	126 (24%)	56 (20%)	17 (28%)	
Symptoms of depression					0.2723					0.0181
None to mild	1437 (84%)	1312 (81%)	614 (82%)	145 (80%)		525 (80%)	390 (73%)	216 (77%)	42 (71%)	
Moderate/Severe	283 (16%)	304 (19%)	137 (18%)	36 (20%)		129 (20%)	145 (27%)	64 (23%)	17 (29%)	
Trust in government entities					<0.0001					<0.0001
No	226 (13%)	561 (35%)	199 (27%)	41 (22%)		95 (14%)	218 (41%)	81 (29%)	14 (23%)	
Yes	1499 (87%)	1060 (65%)	552 (74%)	142 (78%)		561 (86%)	318 (59%)	199 (71%)	46 (77%)	
Trust in healthcare providers					<0.0001					<0.0001
No	425 (25%)	656 (40%)	232 (31%)	65 (36%)		183 (28%)	294 (55%)	102 (36%)	25 (42%)	
Yes	1300 (75%)	965 (60%)	519 (69%)	118 (64%)		473 (72%)	242 (45%)	178 (64%)	35 (59%)	
Health literacy					0.071					0.1498
No	96 (6%)	105 (6%)	42 (6%)	16 (9%)		42 (6%)	46 (9%)	22 (8%)	6 (10%)	
Yes	1556 (90%)	1414 (87%)	670 (89%)	159 (87%)		590 (90%)	459 (86%)	239 (85%)	53 (88%)	
Perceived worry about COVID-19					<0.0001					<0.0001
Not worried	790 (46%)	925 (57%)	438 (58%)	86 (47%)		257 (39%)	286 (53%)	141 (50%)	23 (38%)	
Worried	935 (54%)	696 (43%)	313 (42%)	97 (53%)		399 (61%)	250 (47%)	139 (50%)	37 (62%)	

1: Endorsed safety of both flu and COVID-19 vaccines during pregnancy; 2: endorsed safety of neither flu nor COVID-19 vaccine during pregnancy; 3: endorsed only safety of flu vaccine during pregnancy; 4: endorsed only safety of COVID-19 vaccine during pregnancy.

Among women of reproductive age, similar patterns were observed for most characteristics. While beliefs about the safety of vaccinations during pregnancy did not differ significantly by age group in this subsample, attitudes varied by several other factors, including whether there were children in the household and symptoms of anxiety and depression. Participants who endorsed the safety of both vaccines during pregnancy and those who endorsed the safety of only the COVID-19 vaccine during pregnancy were more likely to not have children in the household (53% and 60%, respectively) compared to those who endorsed the safety of neither vaccine or only the influenza vaccine during pregnancy. Higher proportions of participants reported moderate to severe symptoms of anxiety and depression among those who endorsed the safety of either the COVID-19/influenza vaccine only or neither vaccine compared to those who endorsed the safety of both vaccines during pregnancy (20–28% vs. 16%).

### 3.3. Correlates of Perceived Safety of Only the Influenza Vaccine (vs. Both Vaccines) During Pregnancy

**Figure 1:**
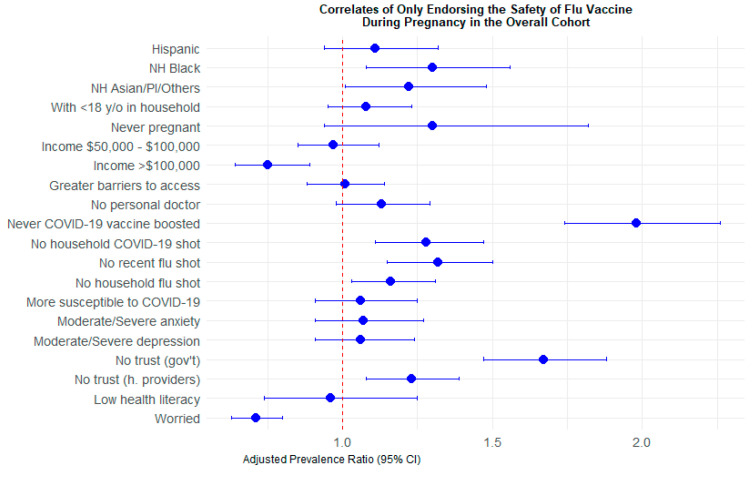
Adjusted prevalence ratios and CIs of only endorsing the safety of the flu vaccine (vs. endorsing the safety of both vaccines) during pregnancy in the overall cohort.

Non-Hispanic Black adults in the overall cohort (aPR: 1.30, 95% CI: 1.08–1.56) were significantly more likely than non-Hispanic White adults to endorse the safety of only the influenza vaccine (vs. both vaccines) during pregnancy (Table 3, Figure 1). Individual and household vaccination behaviors were associated with safety perceptions of the COVID-19 and influenza vaccines during pregnancy. More specifically, not having received a COVID-19 booster (aPR: 1.98, 95% CI: 1.74–2.26), living in a household where no one was vaccinated for COVID-19 (aPR: 1.28, 95% CI: 1.11–1.47), not having recently received an influenza vaccine (aPR: 1.32, 95% CI: 1.15–1.50) and living in household where no one was vaccinated for influenza (aPR: 1.16, 95% CI: 1.03–1.31) were all associated with higher prevalences of endorsing the safety of the influenza vaccine only compared to endorsing the safety of both vaccines during pregnancy. Participants in the overall cohort who did not endorse trust in government entities (aPR 1.67, 95% CI: 1.47–1.88) and healthcare providers (aPR: 1.23, 95% CI: 1.08–1.39) were more likely to endorse only the safety of the influenza vaccine during pregnancy compared to those who endorsed the safety of both vaccines during pregnancy.

**Table 3:** Correlates of only endorsing the safety of the flu vaccine among pregnant women.

Endorsing Only the Safety of the Influenza Vaccine vs. Endorsing the Safety of Both Vaccines for Pregnant Women				
	Overall Cohort		Women of Reproductive Age	
Total	Crude PR (95% CI)	aPR (95% CI)	Crude PR (95% CI)	aPR (95% CI)
Age				
18–29	1.08 (0.91–1.28)		0.95 (0.76–1.19)	
30–39	REF		REF	
40–49	1.04 (0.86–1.26)		0.94 (0.72–1.21)	
50–64	1.29 (1.09–1.54)			
65+	1.49 (1.23–1.80)			
Gender				
Male	0.94 (0.83–1.06)			
Female/non-binary	REF			
Race/Ethnicity				
Hispanic	1.15 (0.97–1.36)	1.11 (0.94–1.32)	1.30 (1.01–1.67)	1.15 (0.89–1.48)
Non-Hispanic White	REF	REF	REF	REF
Non-Hispanic Black	1.46 (1.22–1.76)	1.30 (1.08–1.56)	1.47 (1.08–2.01)	1.26 (0.92–1.72)
Non-Hispanic Asian/PI/other	1.14 (0.94–1.38)	1.22 (1.01–1.48)	1.25 (0.94–1.65)	1.26 (0.96–1.66)
Education				
Less than high school/high school	1.58 (1.32–1.90)		1.71 (1.29–2.25)	
Some college	1.49 (1.31–1.70)		1.56 (1.26–1.94)	
College graduate	REF		REF	
Any <18 years in household				
No	REF	REF	REF	REF
Yes	1.07 (0.95–1.21)	1.08 (0.95–1.23)	1.30 (1.06–1.58)	1.16 (0.94–1.43)
Ever pregnant				
Yes	REF	REF	REF	REF
No	1.43 (1.04–1.97)	1.30 (0.94–1.82)	1.44 (1.03–2.01)	1.40 (1.00–1.96)
Household annual income				
<$50,000/Unknown	REF	REF	REF	REF
$50,000–$100,000	0.89 (0.77–1.02)	0.97 (0.85–1.12)	0.91 (0.73–1.14)	1.01 (0.80–1.27)
>$100,000	0.67 (0.57–0.78)	0.75 (0.64–0.89)	0.66 (0.51–0.85)	0.74 (0.57–0.97)
Healthcare access				
Greater barriers to access	0.98 (0.87–1.11)	1.01 (0.88–1.14)	1.00 (0.82–1.22)	0.93 (0.77–1.14)
Fewer barriers to access	REF	REF	REF	REF
Has a personal doctor/provider				
Yes	REF	REF	REF	REF
No	1.07 (0.94–1.23)	1.13 (0.98–1.29)	1.28 (1.05–1.57)	1.32 (1.07–1.61)
Ever boosted for COVID-19				
No	2.14 (1.89–2.42)	1.98 (1.74–2.26)	2.40 (2.00–2.88)	2.16 (1.74–2.67)
Yes	REF	REF	REF	REF
Has a household member who ever received a COVID-19 vaccination				
No	1.37 (1.19–1.57)	1.28 (1.11–1.47)	1.88 (1.54–2.30)	1.73 (1.41–2.12)
Yes	REF	REF	REF	REF
Recently received the flu vaccination				
No	1.36 (1.20–1.55)	1.32 (1.15–1.50)	1.81 (1.50–2.19)	1.65 (1.35–2.01)
Yes	REF	REF	REF	REF
Has a household member who ever received a flu vaccination				
No	1.20 (1.06–1.35)	1.16 (1.03–1.31)	1.31 (1.07–1.60)	1.28 (1.05–1.56)
Yes	REF	REF	REF	REF
Susceptible to COVID -19				
Less	REF	REF	REF	REF
More	1.31 (1.14–1.49)	1.06 (0.91–1.25)	1.54 (1.17–2.02)	1.37 (1.03–1.81)
Symptoms of anxiety				
None to mild	REF	REF	REF	REF
Moderate/Severe	1.12 (0.95–1.32)	1.07 (0.91–1.27)	1.21 (0.95–1.53)	1.08 (0.85–1.38)
Symptoms of depression				
None to mild	REF	REF	REF	REF
Moderate/Severe	1.09 (0.94–1.27)	1.06 (0.91–1.24)	1.14 (0.90–1.43)	1.04 (0.83–1.31)
Trust in government entities				
No	1.74 (1.54–1.97)	1.67 (1.47–1.88)	1.76 (1.44–2.15)	1.61 (1.32–1.98)
Yes	REF	REF	REF	REF
Trust in healthcare providers				
No	1.24 (1.09–1.40)	1.23 (1.08–1.39)	1.31 (1.07–1.60)	1.17 (0.96–1.44)
Yes	REF	REF	REF	REF
Health literacy				
No	1.01 (0.78–1.31)	0.96 (0.74–1.25)	1.19 (0.84–1.70)	1.03 (0.72–1.48)
Yes	REF	REF	REF	REF
Perceived worry about COVID-19				
Not worried	REF	REF	REF	REF
Worried	0.70 (0.62–0.79)	0.71 (0.63–0.80)	0.73 (0.60–0.89)	0.73 (0.60–0.89)

Among women of reproductive age, results were generally similar to the overall cohort. However, race and ethnicity were not significantly associated with safety perceptions of vaccines during pregnancy. A few variables, which were not significant in the overall cohort, were associated with endorsing the safety of only the influenza vaccine versus both vaccines during pregnancy. Not having a personal doctor (aPR: 1.32, 95% CI: 1.07–1.61) and greater susceptibility to COVID-19 (aPR: 1.37, 95% CI: 1.03–1.81) were associated with higher prevalence of endorsing only the safety of the influenza vaccine during pregnancy compared to endorsing both vaccines.

### 3.4. Correlates of Endorsing the Safety of Neither Vaccine (vs. Both Vaccines) During Pregnancy

**Figure 2:**
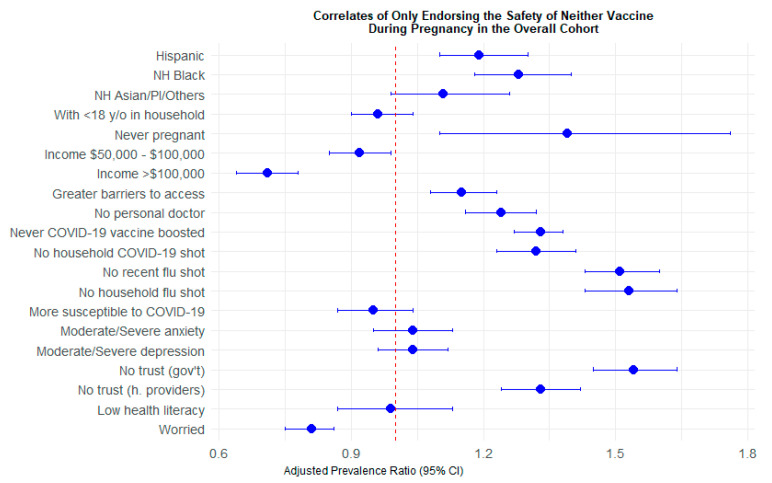
Adjusted prevalence ratios of only endorsing the safety of neither vaccine (vs. endorsing the safety of both vaccines) during pregnancy in the overall cohort.

Patterns were largely similar when assessing the perceived safety of only the influenza vaccine (vs. both) during pregnancy and for endorsing the safety of neither (vs. both) during pregnancy. Hispanic (aPR: 1.19, 95% CI: 1.10–1.30) and non-Hispanic Black adults (aPR: 1.28, 95% CI: 1.18–1.40) were significantly more likely to not endorse the safety of either vaccine during pregnancy compared to White adults (Table 4, Figure 2). Adults who never reported a pregnancy during the study (aPR: 1.39, 95% CI: 1.10–1.76), those with greater barriers to healthcare access (aPR: 1.15, 95% CI: 1.08–1.23), and those who did not have a personal doctor (aPR: 1.24, 95% CI: 1.16–1.32) had higher prevalences of not endorsing the safety of either vaccine than endorsing both vaccines during pregnancy. Participants who had not received a COVID-19 booster (aPR: 1.33, 95% CI: 1.27–1.38), were not in COVID-19-vaccinated households (aPR: 1.32, 95% CI: 1.23–1.41), had not recently received an influenza shot (aPR: 1.51, 95% CI: 1.43–1.60) and were not in influenza-vaccinated households (aPR: 1.53, 95% CI: 1.43–1.64) were significantly more likely to not endorse the safety of either vaccine (vs. both) during pregnancy (Table 4). Adults who did not trust government entities (aPR: 1.54, 95% CI: 1.45–1.64) and did not trust healthcare providers (aPR: 1.33, 95% CI: 1.24–1.42) also had a higher prevalence of not endorsing the safety of either vaccine (vs. both) during pregnancy.

**Table 4:** Correlates of endorsing the safety of neither vaccine among pregnant women.

Endorsing the Safety of Neither Vaccine vs. Endorsing the Safety of Both Vaccines for Pregnant Women				
	Overall Cohort		Women of Reproductive Age	
Total	Crude PR (95% CI)	aPR (95% CI)	Crude PR (95% CI)	aPR (95% CI)
Age				
18–29	0.94 (0.84–1.05)		0.91 (0.78–1.05)	
30–39	REF		REF	
40–49	1.16 (1.05–1.29)		1.08 (0.93–1.26)	
50–64	1.20 (1.09–1.33)			
65+	1.23 (1.10–1.39)			
Gender				
Male	1.00 (0.93–1.07)			
Female/non-binary	REF			
Race/Ethnicity				
Hispanic	1.30 (1.19–1.41)	1.19 (1.10–1.30)	1.57 (1.35–1.83)	1.23 (1.07–1.42)
Non-Hispanic White	REF	REF	REF	REF
Non-Hispanic Black	1.53 (1.40–1.67)	1.28 (1.18–1.40)	1.87 (1.61–2.19)	1.36 (1.17–1.57)
Non-Hispanic Asian/PI/other	1.05 (0.93–1.19)	1.11 (0.99–1.26)	1.18 (0.96–1.46)	1.20 (0.99–1.45)
Education				
Less than high school/high school	1.69 (1.54–1.84)		2.36 (2.03–2.73)	
Some college	1.55 (1.44–1.67)		2.03 (1.76–2.34)	
College graduate	REF		REF	
Any person <18 years in household				
No	REF	REF	REF	REF
Yes	0.99 (0.92–1.07)	0.96 (0.90–1.04)	1.20 (1.05–1.26)	0.96 (0.86–1.08)
Ever pregnant				
Yes	REF	REF	REF	REF
No	1.60 (1.26–2.01)	1.39 (1.10–1.76)	1.51 (1.18–1.92)	1.28 (1.01–1.61)
Household annual income				
<$50,000/unknown	REF	REF	REF	REF
$50,000–$100,000	0.83 (0.76–0.89)	0.92 (0.85–0.99)	0.76 (0.67–0.88)	0.92 (0.81–1.04)
>$100,000	0.61 (0.56–0.68)	0.71 (0.64–0.78)	0.38 (0.30–0.47)	0.48 (0.38–0.61)
Healthcare access				
Greater barriers to access	1.18 (1.10–1.26)	1.15 (1.08–1.23)	1.30 (1.14–1.48)	1.11 (0.99–1.25)
Fewer barriers to access	REF	REF	REF	REF
Has a personal doctor/provider				
Yes	REF	REF	REF	REF
No	1.23 (1.14–1.32)	1.24 (1.16–1.32)	1.42 (1.26–1.61)	1.36 (1.23–1.51)
Ever boosted for COVID-19				
No	2.01 (1.90–2.13)	1.33 (1.27–1.38)	2.51 (2.25–2.80)	1.94 (1.69–2.23)
Yes	REF	REF	REF	REF
Has a household member who ever received a COVID-19 vaccination				
No	1.46 (1.36–1.57)	1.32 (1.23–1.41)	1.88 (1.68–2.10)	1.46 (1.31–1.64)
Yes	REF	REF	REF	REF
Recently received the flu vaccination				
No	1.88 (1.76–2.01)	1.51 (1.43–1.60)	2.66 (2.32–3.05)	2.18 (1.88–2.54)
Yes	REF	REF	REF	REF
Has a household member who ever received a flu vaccination				
No	1.66 (1.55–1.78)	1.53 (1.43–1.64)	2.01 (1.78–2.28)	1.71 (1.51–1.94)
Yes	REF	REF	REF	REF
Susceptible to COVID-19				
Less	REF	REF	REF	REF
More	1.15 (1.06–1.25)	0.95 (0.87–1.04)	1.42 (1.21–1.67)	1.11 (0.96–1.28)
Symptoms of anxiety				
None to mild	REF	REF	REF	REF
Moderate/severe	1.10 (1.00–1.21)	1.04 (0.95–1.13)	1.28 (1.12–1.48)	1.07 (0.95–1.21)
Symptoms of depression				
None to mild	REF	REF	REF	REF
Moderate/severe	1.09 (0.99–1.18)	1.04 (0.96–1.12)	1.24 (1.09–1.42)	1.05 (0.94–1.18)
Trust in government entities				
No	1.72 (1.61–1.83)	1.54 (1.45–1.64)	1.93 (1.72–2.16)	1.53 (1.36–1.71)
Yes	REF	REF	REF	REF
Trust in healthcare providers				
No	1.42 (1.33–1.52)	1.33 (1.24–1.42)	1.82 (1.61–2.06)	1.44 (1.27–1.64)
Yes	REF	REF	REF	REF
Health literacy				
No	1.10 (0.96–1.26)	0.99 (0.87–1.13)	1.19 (0.97–1.48)	0.94 (0.78–1.13)
Yes	REF	REF	REF	REF
Perceived worry about COVID-19				
Not worried	REF	REF	REF	REF
Worried	0.79 (0.74–0.85)	0.81 (0.75–0.86)	0.73 (0.65–0.83)	0.77 (0.69–0.86)

Among women of reproductive age, similar associations were found, except that greater barriers to healthcare were not significantly associated with a lower endorsement of the safety of either vaccine (vs. both) during pregnancy (Table 4).

## 4. Discussion

In this study of perceptions of the safety of the COVID-19 and influenza vaccines during pregnancy, less than half (43%) of all adult participants believed that COVID-19 vaccines were safe during pregnancy, and just over half (55%) believed the influenza vaccine to be safe during pregnancy. While 40% perceived both vaccines to be safe during pregnancy, over a third of the cohort (38%) did not endorse the safety of either vaccine during pregnancy, while 18% perceived the influenza vaccine to be safe during pregnancy but remained neutral or negative regarding the safety of the COVID-19 vaccine during pregnancy. These findings highlight that the influenza vaccine appears to have substantially greater perceived safety during pregnancy than the COVID-19 vaccine in this sample. Significant work needs to be conducted to increase awareness, understanding, and trust in the safety and importance of vaccinations during pregnancy to protect the health of pregnant people and their infants.

Our study underscores the ongoing and potentially increasing gap in perceptions about vaccine safety during pregnancy. Long before the COVID-19 pandemic, the ACOG has been advising that everyone who is or will be pregnant during influenza season should receive an annual influenza vaccine as soon as it becomes available each year [32] and has recommended receipt of the COVID-19 vaccine during pregnancy since August of 2021 [33]. While individuals who have not previously been pregnant are unlikely to have received counseling from a specialist in obstetrics and gynecology or a midwife about the safety of either vaccine during pregnancy, even among women of reproductive age, only 18% endorsed the safety of the influenza vaccine during pregnancy and just 4% endorsed the safety of the COVID-19 vaccine during pregnancy, suggesting large gaps in vaccine safety perceptions among a potentially relevant group.

It is important to consider the unique challenges regarding COVID-19 communications and messaging around pregnancy during the pandemic, from the initial unknowns about the threat of COVID-19 to frequent and rapidly changing guidelines for prenatal care [34]. Studies focusing on pregnant women’s COVID-19 vaccine decision-making processes have stressed how complex and conflicting a decision this has been for many, while also highlighting the effects of emotional factors associated with vaccination decision-making processes, cultural perspectives, and the influence of trusted supporters [35,36]. Prior studies focusing on influenza vaccination among pregnant women found that recommendations from healthcare professionals greatly increased the odds of vaccination. However, believing there is a potential of vaccine-induced harm negatively affected the odds of vaccination to a similar degree [37]. This suggests a critical gap in the existing communication strategies for promoting the safety of both the COVID-19 and influenza vaccines during pregnancy, for both pregnant women and the community of trusted supporters around them, underscoring the importance of broader adult attitudes in creating a supportive environment conducive to vaccination during pregnancy.

We examined factors associated with discordant and concordant vaccine safety perceptions among adults regarding the influenza and COVID-19 vaccines during pregnancy. Our findings highlight that race and ethnicity, trust in institutions, and healthcare access play key roles in these vaccine safety perceptions. Individuals identifying as non-Hispanic Black or non-Hispanic Asian/Pacific Islander/other, those lacking trust in government entities, and those lacking trust in healthcare providers were more likely to endorse only the influenza vaccine as safe during pregnancy. Non-Hispanic Black respondents, Hispanic individuals, and those who distrusted both government entities and healthcare providers or faced healthcare access barriers were less likely to endorse the safety of either vaccine during pregnancy. Among women of reproductive age, distrust in government entities was associated with endorsing only the influenza vaccine as safe, while Hispanic respondents and those with low trust in both government and healthcare providers had a higher prevalence of perceiving neither vaccine as safe during pregnancy.

Research indicates that people’s lack of trust in the government and scientists is strongly associated with vaccine hesitancy, a trend that predates the COVID-19 pandemic [38,39,40]. A 2018 study found that individuals with lower trust in healthcare providers and government medical experts had less intention to vaccinate against the flu, pertussis, and measles [41]. In the context of the recent pandemic, global studies have demonstrated a negative correlation between trust in science and COVID-19 vaccine hesitancy, while trust in government information has also been shown to reduce vaccine hesitancy [42]. Some suggest that trust in institutions like the government and healthcare providers increases the likelihood of believing their messages [43] and that trust in these entities is interdependent [44], while distrust can lead individuals to believe opposing information or even view vaccine refusal as a political act of resistance [45,46]. Hence, building trust in the government and healthcare providers is a challenging yet essential task to combat vaccine hesitancy for COVID-19 and other vaccine-preventable diseases.

On another note, the recent literature has given less attention to the role of healthcare access in shaping vaccine perceptions. Studies have shown that individuals with health insurance, a primary care provider, and recent routine checkups are less likely to be vaccine-hesitant [47,48]. These relationships extend to pregnant women, indicating that healthcare providers play a pivotal role in influencing vaccination decisions [49,50,51]. However, barriers such as unclear vaccine guidelines for pregnant women, time constraints and multiple appointments, and cost can influence providers’ recommendations [49,51,52]. Improving healthcare access, addressing systemic inequities, and enhancing provider education and support are critical strategies for reducing barriers to healthcare access and increasing vaccination rates.

Consistently, our results show that having a personal doctor, a prior pregnancy, prior influenza vaccination, and prior COVID-19 vaccination were associated with perceiving both vaccines as safe during pregnancy. This highlights that personal experience with the medical system is associated with vaccine perceptions. Similarly, observing peers get vaccinated can alleviate psychological uncertainty and enhance perceived vaccine safety, highlighting the action cues from others in the community, including doctors, friends and family, country leaders, and community leaders [53]. These findings underscore the importance of fostering trust in the medical system, reducing access barriers, and leveraging community-driven messaging to promote trust in the safety of vaccines during pregnancy.

### Limitations

This study has several limitations. First, the analysis used data collected at the end of 2023, which may limit the relevancy in this evolving landscape of vaccine uptake and messaging. Perceptions of vaccine safety during pregnancy may have changed since the time of data collection. However, there is no recent evidence to suggest that vaccine perceptions have improved since 2023. As an online cohort, self-reported data may introduce recall and social desirability bias. However, the short interval between follow-up assessments may reduce recall bias. We also expect minimal social desirability bias as misclassification is limited from the dichotomization of responses.

While dichotomization of certain Likert responses may result in loss of information, our primary research question was to describe and explore concordance and discordance across vaccine types. Dichotomization also increased the analytic ability to discern proactive agreement and create four distinct categories. Data on vaccine safety perceptions were collected at a single time point, limiting the ability to establish temporality or causal relationships. The findings are descriptive and exploratory rather than causal. Additionally, no formal adjustments for multiple comparisons across endorsement patterns were applied; therefore, the findings should be interpreted with caution due to the potential of Type I error.

This study captured respondent sentiment about the safety of the influenza and COVID-19 vaccines during pregnancy but is not limited to those who have had to make the decision to get vaccinated during pregnancy. While there may be additional factors most relevant to this group, understanding the beliefs of the wider adult population is essential for developing effective strategies that foster a supportive environment for vaccination during pregnancy. Additionally, we did not examine participants who endorsed the safety of the COVID-19 vaccine during pregnancy but not the influenza vaccine because of the small sample size of this group. There may be particular factors that distinguish this group as well. Further investigation is needed to determine whether our findings apply to other vaccines recommended during pregnancy, such as Tdap and MMR.

## 5. Conclusions

Perceptions of vaccine safety during pregnancy remain low among US adults and among US women of reproductive age, particularly for COVID-19 vaccines, with substantial proportions of respondents reporting uncertainty or negative perceptions about both COVID-19 and influenza vaccines during pregnancy. Our findings highlight the important role that trust in institutions, healthcare access, and prior engagement with the healthcare system play in shaping these perceptions. They also highlight that broader public perceptions about vaccine safety may shape the social environment in which vaccination decisions during pregnancy are made.

These results underscore the need for multi-level strategies to improve the perception of the COVID-19 and flu vaccines as safe to receive during pregnancy. Healthcare providers, including obstetricians, midwives, and primary care clinicians, are critical sources of trusted information and should be supported through training, clear guidance, and sufficient time for patient counseling. Community-based approaches may also help address vaccine concerns by engaging trusted messengers such as community and religious leaders and leveraging peer networks to reinforce accurate information about vaccine safety during pregnancy. At the policy level, efforts to strengthen healthcare access, support consistent public health messaging, and address institutional trust, particularly among communities historically underserved by the healthcare system, may help improve confidence in the safety of vaccination during pregnancy and reduce disparities in vaccine uptake.

## Data Availability

Study data from the CHASING COVID Cohort is publicly available on Zenodo (DOI: 10.5281/zenodo.6127734). Some data elements and surveys from the CHASING COVID Cohort study are not made publicly available due to funder requirements, but this information can be made available to researchers with approved projects.
